# Ultrasonography Findings in the Proximal Sciatic Nerve and Deep Gluteal Muscles in 29 Dogs With Suspected Sciatic Neuritis

**DOI:** 10.3389/fvets.2021.704904

**Published:** 2021-08-27

**Authors:** Tiiu M. Toijala, Debra A. Canapp, Sherman O. Canapp

**Affiliations:** ^1^Sporting Dogs Clinic Evidensia, Espoo, Finland; ^2^Veterinary Orthopedic and Sports Medicine Group, Annapolis Junction, MD, United States

**Keywords:** sciatic nerve, ultrasonography, dog, sciatic neuritis, deep gluteal muscles

## Abstract

The present study aimed to describe the ultrasonography technique and analyze the ultrasonographic findings of the proximal sciatic nerve and deep gluteal muscles in dogs with suspected sciatic neuritis. The records of 29 dogs that underwent musculoskeletal ultrasound of the sciatic nerve and deep gluteal muscles were retrospectively evaluated. Both right and left sides were evaluated. Abnormal findings were unilateral in 28/29 (97%) of the dogs. The muscles examined included the piriformis muscle, gemelli muscles, internal obturator muscle, and medial gluteal muscle. Data included signalment, purpose of the dog, orthopedic examination findings, lameness examination findings, other diagnostic imaging findings, and ultrasonography findings. Irregular margins of the sciatic nerve were recorded in 76% of the dogs. The diameter of the sciatic nerve on the affected limb was significantly larger than the nerve on the contralateral, unaffected side (*p* < 0.00001). The mean ± standard deviation (SD) of the sciatic nerve inner diameter between the unaffected and affected limbs was 15 ± 14.66%. The mean ± SD in sciatic nerve outer diameter was 12 ± 7.71%. Abnormal ultrasonographic findings in at least one of the examined muscles were found in 28/29 (97%) of the dogs and included changes in echogenicity in 28/29 (97%) of the dogs, loss of detail in muscle fiber pattern in 5/29 (17%) of the dogs, and impingement between the sciatic nerve and piriformis muscle in 6/29 (21%) of the dogs. The most common underlying diagnosis was lumbosacral stenosis in 9/29 (31%) of the dogs. The most common sport was agility for 21/29 (71%) of the dogs. Repetitive jumping may predispose to both lumbosacral disease, through dynamic compression, and changes within the piriformis muscle, due to overuse of the muscle leading to irritation to sciatic nerve. Ultrasonography is considered a safe and non-invasive diagnostic method to evaluate the sciatic nerve and deep gluteal muscles of dogs, and provides additional guidance for diagnostics and rehabilitation planning. This is the first study documenting ultrasonography findings in a case series of the sciatic nerve and deep gluteal muscles and their pathology in dogs.

## Introduction

Sciatic neuritis, commonly referred to as sciatica, is a relatively common pathological condition in humans. It has been estimated that approximately 5–10% of patients with lower back pain have sciatica. The underlying conditions typically resulting in sciatic neuritis in humans include lumbosacral diseases, tumors compressing the sciatic nerve, and deep gluteal syndrome (DGS) or piriformis syndrome (1–6). Degenerative disc disease or lumbar disc herniation (LDH) compressing the lumbosacral nerve roots is the most common cause of sciatic nerve irritation (2). Approximately 5% of sciatic cases in humans are extraspinal in origin (1). Deep gluteal syndrome (DGS) is an entity characterized by pain in the buttock area, hip, or posterior thigh and/or radicular pain due to non-discogenic sciatic nerve entrapment in the subgluteal space. Symptoms may include piriformis and obturator internus/gemellus syndromes, quadratus femoris/ischiofemoral pathology, hamstring conditions, and gluteal disorders. Although there is often pathology in the surrounding structures, DGS is frequently referred to as piriformis syndrome (6). Piriformis syndrome is an elusive condition that is likely to be overlooked and overdiagnosed in equal proportions. The piriformis muscle may be irritated in either conjunction with other structures or secondary to other disorders such as hip or sacroiliac joint disease (1).

Interestingly, a case report described sciatic neuritis as being caused by an adductor muscle strain in a human patient (7). The adductor muscle tendon insertion runs adjacent to the sciatic nerve. Because of this proximity between the sciatic nerve and the adductor muscle, strains of this tendon may also be a possible underlying cause in dogs with sciatic neuritis. In humans, there are anatomical variations in the sciatic nerve. There have been contradictions regarding the statistical significance of the relationship between sciatic nerve anatomical variation and the clinical diagnosis of sciatica in humans (5, 8). The authors of the current article are not familiar with studies on anatomical variations of the sciatic nerve in dogs. However, one study investigated the anatomy of the sciatic nerve in four dogs, and no structural differences were noted (9).

Sciatic neuritis in humans is often explained and understood to be caused by nerve compression: while this is often part of the pathological process, inflammation, and neural sensitization also play important roles (10). The compression of the nerve can be located at the level of the nerve roots or more distally in the area in which deep gluteal muscles surround the sciatic nerve.

In dogs, the sciatic nerve may be injured due to injections into the hind leg, mechanical trauma such as femoral fractures, tumors, or as a complication in a surgery (11). There is also a case study describing a sciatic neuropathy in two dogs after a spontaneous bleeding causing muscular and intraneural hemorrhage (12). These incidents may cause substantial damage to the nerve and in most cases neurologic deficits. Lumbosacral foraminal stenosis in dogs can cause radicular pain, and changes in electrodiagnostics in the tibial nerve, a branch of the sciatic nerve (13, 14).

Diagnoses of neuritis in dogs can be suspected based on clinical findings and confirmed by electrodiagnostics (electromyography) or diagnostic imaging tools, such as magnetic resonance imaging (MRI) and ultrasonography (4, 5). Electromyography and MRI are the most commonly used diagnostic tools for sciatic neuritis and the underlying issues in the lumbosacral region. Lumbosacral nerve root compression can affect motor and sensory nerve conduction in canines, which is detected using electrodiagnostics (13). However, there are limitations to electromyographic studies: some human studies have suggested that electromyographic changes can diminish over time. The optimal window for performing electromyography is approximately 3 weeks after the onset of signs (15). Ultrasonography has recently become recognized as an effective alternative diagnostic tool, particularly in human and equine sports medicine. Ultrasonography allows for diagnosis as well as immediate interventional guidance if injections to the area are required. Unlike other diagnostic modalities, ultrasonography allows dynamic imaging, which allows specific evaluation of nerve gliding with possible impingement from the surrounding muscles. Studies in humans comparing electromyography to ultrasound have concluded that ultrasound is a more valid and accurate diagnostic modality (16, 17). Ultrasound has been proven to be beneficial for evaluating nerves in humans (18–21). The main pathological feature that has been demonstrated by MRI or ultrasonography is nerve enlargement (19, 22), and several studies in human patients with sciatica have shown that the sciatic nerve is enlarged on the side of the sciatica (23, 24). In dogs, diagnostic ultrasound has been used to locate nerves to perform nerve blocks (25, 26). It can be performed either without sedation or under light sedation. Since complete anesthesia is not needed to perform an evaluation, it is safer and is considered a less invasive procedure than MRI. Moreover, it is a less expensive procedure for owners. These advantages also make ultrasound a viable option for subsequent reassessments for the tissue healing. Metal implants can be problematic with MRI due to the strong magnetic field in MRI. Ultrasound is not affected by metal implants outside the area to be scanned, therefore making it more appealing than MRI.

To best of our knowledge, there have been two studies describing the ultrasonographic assessment of the sciatic nerve in dogs (14, 27). One described the ultrasonography technique for sciatic nerve along the entire length of the nerve in four cadavers, two live dogs, and five patients (14). The other study described ultrasonography findings including measurements of the diameter of the sciatic nerve before and after dissection of the tibial nerve in 7 dogs (27). However, the ultrasonography transducer used in this study was 7.5 mHz, providing only moderate resolution to the image. Technology regarding ultrasound machines has since evolved, providing much better resolution of the tissues. We believe it is important to identify and describe the findings with a higher resolution transducer, as these provide more detailed information and are widely available for practitioners today. The transducer used in our study was 18 MHz linear transducer.

To be able to perform and interpret the findings of the ultrasound examination, it is important to be familiar with the anatomy and function of the muscles around the sciatic nerve. The piriformis muscle lies deep to the middle gluteal muscle and the mid-caudal region is covered by the superficial gluteal muscle. The piriformis muscle arises on the lateral surface of the third sacral and first caudal vertebrae. In addition, the tendon of insertion joins that of the musculus gluteus medius on the greater trochanter of the femur. The function of the piriformis muscle is to extend the hip joint. The piriformis muscle is innervated by the caudal gluteal nerve (28). The sciatic nerve is found caudal and deep into the piriformis muscle.

**Figure 1:**
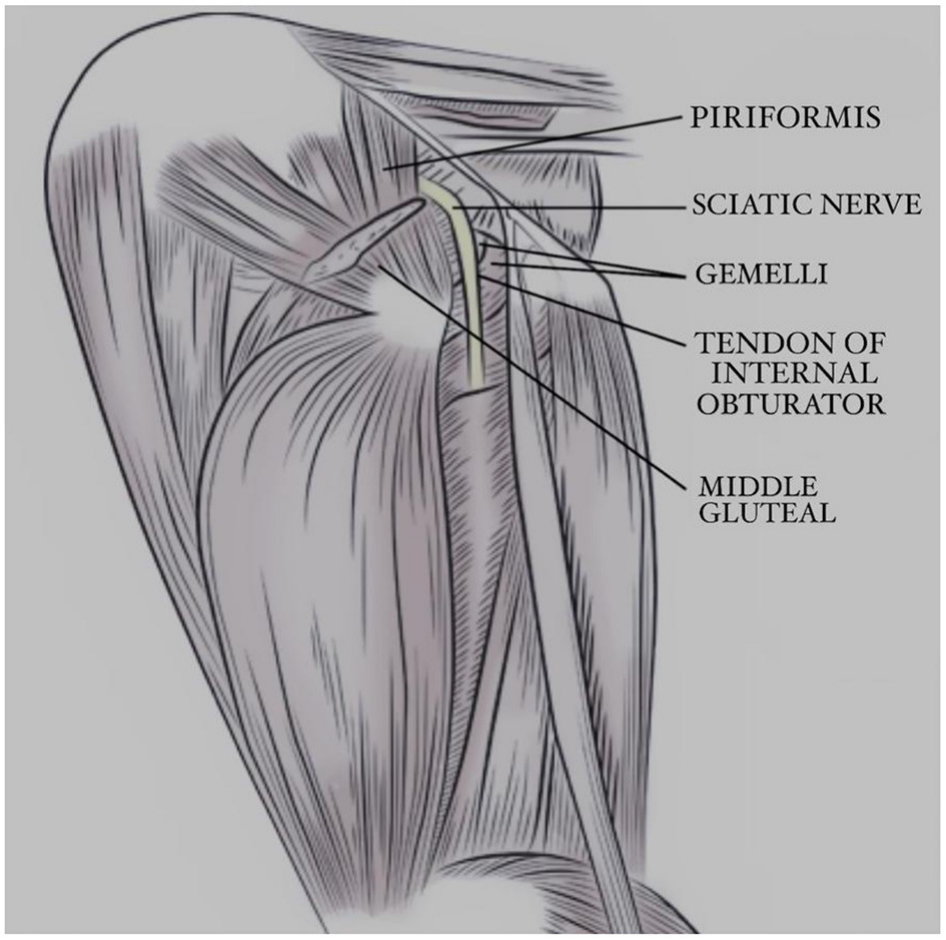
Illustration of the anatomy of the deep gluteal region in a dog, lateral view. Courtesy by Clean Run Magazine.

**Figure 2:**
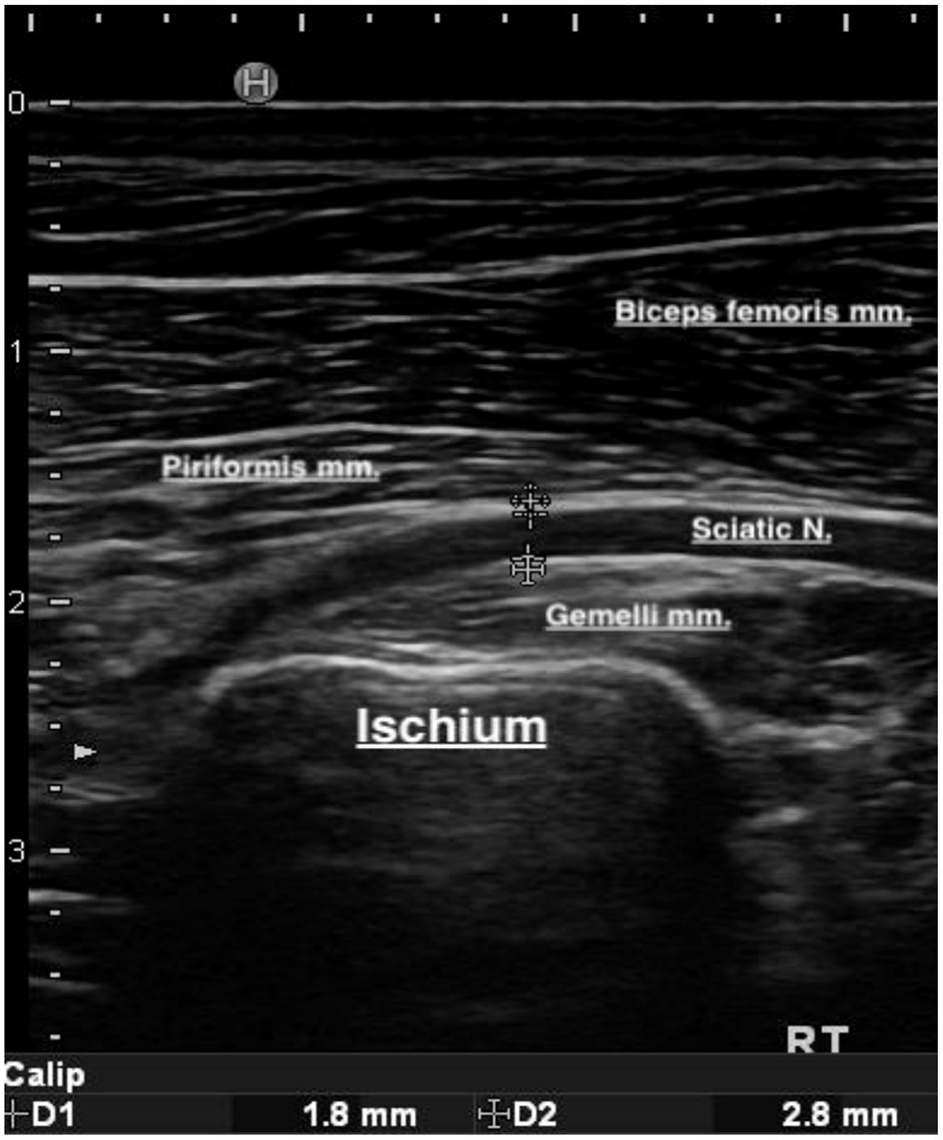
Linear ultrasonography image of the sciatic nerve and the surrounding muscles. The inner and outer margins of the sciatic nerve have been measured. The inner margin (D1) is 1.8 mm, and the outer margin (D2) 2.8 mm.

The canine internal obturator muscle is fan-shaped and possesses great strength. It arises medially to the obturator foramen on the pelvic surfaces of the rami of the pubis and ischium, the ischiatic table, and from the ischiatic arch. It passes over the smooth surface of the lesser ischiatic notch directly caudal to the ischiatic spine and ventral to the sacrotuberous ligament. The muscle ends as a flat tendon between the two gemelli muscles. Overhanging portions of the gemelli muscles run from the edge of the lesser ischiatic notch into the internal obturator tendon, ending in the trochanteric fossa. The function of the gemelli muscle is to perform lateral rotation of the hip joint and prevent medial rotation due to weightbearing. The sciatic nerve runs superficially to these structures (See Figures 1, 2). Both the internal obturator and gemelli muscles are innervated by the sciatic nerve (28).

The middle gluteal muscle arises from the iliac crest and most of the tuber sacrale. Some fibers also originate from the dorsal portion of the sacroiliac ligament and from the deep layer of the gluteal fascia. A large part of the muscle lies deep to the gluteal fascia and skin and is caudally covered by the superficial gluteal muscle. In the caudodistal direction, it extends over the musculus gluteus profundus and ends in the trochanter major. A narrow but thick deep belly can be defined from the caudal border of the middle gluteal muscle. This deep portion arises on the transverse processes of the last sacral and first caudal vertebrae, and the sacrotuberous ligament ends on the trochanter major with its tendon. Its action is an extension of the hip joint, medial rotation of the hip, and prevention of lateral rotation during weight-bearing. The medial gluteal muscle is innervated by the caudal gluteal nerve (28).

Because a compression injury often causes neuritis, it is essential to evaluate the surrounding muscles of the nerve during an ultrasound examination, and we therefore included the piriformis muscle, gemelli muscles, internal obturator muscle, and middle gluteal muscles in the musculoskeletal ultrasound examination. The purpose of this study was to describe the ultrasonography technique and discuss the ultrasonographic findings of the sciatic nerve and deep gluteal muscles in 29 dogs with suspected sciatic neuritis.

## Materials and Methods

### Case Selection

Canine patient files were evaluated from 2017 to 2019 for dogs that underwent musculoskeletal ultrasound for the sciatic nerve region in both legs, including the piriformis, middle gluteal, internal obturator, and gemelli muscles at the Veterinary Orthopedic and Sports Medicine Group (VOSM), Annapolis Junction, Maryland, USA. The recommendation for the ultrasound examination had been based on history, physical examination findings, and other diagnostic imaging findings. Breed, weight, age, purpose of the dog, duration of clinical signs, lameness examination findings, physical examination findings, ultrasound findings, radiological findings, magnetic imaging findings (if available), and primary diagnosis were recorded. The purpose of the dog indicated whether the dog was active in any sport or mainly a companion dog. If the dog was involved in sports, the sport was also documented.

### Orthopedic Examination

Complete orthopedic examination, including gait assessment and palpation, was performed by ACVS board-certified surgeons at the VOSM.

Lameness examination was performed subjectively, objectively, or both. Temporospatial gait analysis was used to determine the objective lameness and to obtain length measurements. The investigation involved walking canine patients down a pressure-sensitive walkway (Gait4Dogs, CIR Systems Inc., Franklin, NJ, USA). Walks were considered acceptable if they were within parameters considered using analysis software and were compared to similar walks in terms of gait cycle and velocity. Measurements recorded included total pressure index (TPI%), stride length, step length, and the ratio between stride and step length.

Subjective gait analyses included evaluation of walking and trotting from the front, behind, and both sides. Walking and trotting in circles were also assessed. Gait analyses was graded as follows: no lameness, mild lameness, moderate lameness, or severe lameness. Lameness was considered as follows: mild, if it was in a trot with a trained eye; moderate, if there was a noticeable head bob or lifting of the iliac wing at the trot; and severe, if there is toe touching or non-touching lameness.

Palpation included palpation of the muscles, spine, bones and joints. Muscles included palpation of the neck, back and limbs for symmetry, tightness, pain, atrophy, heat, and swelling. Palpation of the joints included evaluation of range of motion, pain, crepitus, instability, heat, and swelling. Bones were palpated for pain, swelling, and crepitus. Spine was evaluated for pain and stiffness. Any asymmetry or abnormal findings were recorded.

### Evaluation of the Sciatic Nerve and Deep Gluteal Muscles Using Ultrasound

Ultrasonography was performed by a single ACVSMR board-certified practitioner with high level of experience with musculoskeletal ultrasound (DC^2^).

Landmarks to be identified for the sciatic and deep gluteal muscles ultrasound examination include the greater trochanter of the femur and the ischiatic tuberosity. Both legs were examined, and the unaffected leg was used as a normal control.

### Ultrasound Imaging Method

Ultrasound examination was performed with Hitachi-Aloka Noblus system, using an 18 Mhz linear probe. The canine patients were scanned either under general anesthesia or mild sedation or sometimes while conscious, depending on the comfort level. Both right and left sides were scanned using the unaffected side as a reference. The hair was shaved and wiped clean with alcohol. The dogs were placed in lateral recumbency with their hind limbs positioned at a normal standing angle. Ultrasound gel was applied to the probe surface and skin to enhance visualization of the peripheral nerves and musculoskeletal structures of the upper hind extremity. The greater trochanter and ischial tuberosity ridge were palpated and identified and the ultrasound probe was placed longitudinally in between these landmarks, with the marker situated proximally, which corresponds to the left side of the ultrasound screen. The ultrasound probe, placed parallel to the sciatic nerve, was then glided proximally, in addition to a slight fanning of the tail to obtain and identify a clear image of the sciatic nerve.

At this level, the sciatic nerve was identified. The sciatic nerve perineurium produces two distinct, bright boundary echoes, with separate hyperechoic parallel lines and a more hypoechoic center, similar to larger vessels, but flow negative on color Doppler. These findings are similar in cross-sectional images of the sciatic nerve with more defined fascicles and intra-epineurium if the zoom and gain are increased. In addition, at this level and ultrasound probe placement, the distinct hyperechoic margins of the piriformis muscle belly (superficial to the sciatic nerve), the gemelli (deep to the sciatic nerve), and the pelvic bone border could be identified, and the muscles examined in long axis in this plane of view. At this level, the measurements of the inner and outer margins of the nerve were taken from longitudinal images at the time of the ultrasonographic examination, for both the affected and unaffected limb in each patient, and recorded in millimeters (mm). Measurements were taken 2–3 times, and the median of the measurements was recorded.

When examining the middle gluteal muscle, its insertional tendon was first identified by placing the distal end of the ultrasound probe on the greater trochanter and angling the proximal part of the ultrasound probe slightly toward to ilium. Once the insertional tendon was identified, the middle gluteal muscle belly was followed dorso-cranially, approaching the origin margins on the wing of the ilium when possible. When examining the internal obturator tendon and bursa, the ultrasound probe was again placed within the groove between the caudal aspect of the greater trochanter and the ischial wing, where the long axis of the sciatic nerve was identified. The internal obturator tendon was identified on the distal margin with its distinctive bursa/fluid artifact, creating a fluid shadow at the level of the internal obturator tendon and bursa and assessed at the caudal distal margin of the gemelli muscle, all deep to the sciatic nerve.

### Other Diagnostic Modalities

Other diagnostic imaging modalities included radiography and MRI. Radiography was performed for 24/29 cases when there were findings suggestive of either stifle or lumbosacral disease. Radiography was performed using Sound Tru DR DX 1700. Radiographs were read by ACVS board-certified surgeons at VOSM.

Magnetic resonance imaging of the lumbar spine was performed if there was a suspicion of an underlying lumbosacral disease after physical examination and radiology. Magnetic resonance imaging was performed on 10 dogs using the Philips Gyroscan 1.5 Tesla Unit. Magnetic resonance imaging was performed by ACVIM (Neurology) board-certified neurologist at VOSM and images were read by radiologists. Radiographic examinations were based by clinical findings in the dog, and included radiographs of thoracic and lumbar spine, hips, stifles, and tarsus. Magnetic resonance imaging examinations were based by clinical and radiological findings and included lumbar spine examinations. All abnormal findings were recorded.

### Statistics

The data was determined not to be normally distributed based on histogram evaluation. A one-tailed Wilcoxon Signed-Rank test was used to evaluate the difference in inner and outer sciatic nerve diameter between the affected and unaffected limb of each dog, to the alpha = 0.05 significance level.

Sciatic nerve measurements for dogs with clinical evidence of orthopedic disease were compared to measurements from dogs without clinical evidence of orthopedic disease using the Mann-Whitney U test, to the alpha = 0.05 significance level. The variables analyzed were the absolute and percent difference in inner sciatic diameter and outer sciatic diameter between the affected and unaffected limbs.

## Results

### Dogs

The dogs breeds were as follows: Border Collie (*n* = 13; 45%), Boxer, Jack Russell Terrier, Collie, Shetland Sheepdog, Leonberger, mix breed, Nova Scotia Duck Tolling Retriever, Australian Shepherd, Staffordshire Bull Terrier, German Shepherd, Whippet, Golden Retriever, Labrador Retriever, Rat Terrier, and Standard Poodle. The weight of the dogs ranged from 24.4 to 100 pounds, with a mean standard deviation (SD) of 46.5 ±19.3 pounds. The ages of the dogs ranged from 2 to 11 years, with a mean ± SD of 5 ± 2.7 years. The purpose of the dogs was as follows: 21 (72%) dogs were either training, competing, or both in agility, 2 (7%) in obedience, 1 (3%) in field trial, 1 (3%) in schutzhund (IPO), and 4 (14%) were companion dogs. The duration of the clinical signs according to the owner varied from a few days to 3 years, with a mean ±SD of 37 ±10 weeks.

### Orthopedic Examination

#### Gait Assessment

Gait assessment records were available for 26 of the 29 dogs. Gait assessment included either subjective (26 dogs) or subjective and objective gate assessments (21 dogs).

Objective gait assessment findings were available for 21/29 dogs. Total Pressure Index (TPI) was decreased 1% or more compared to the normal contralateral side in 11/17 (65%) dogs on the ipsilateral side of the lameness. Step length was reduced 9% in only one of the 17 dogs and was similar in 4/17 (24%) dogs.

Subjective gait analyses was performed by ACVS board-certified surgeons at the VOSM. Findings included shortened stride in the ipsilateral limb in 21 dogs, mild lameness in nine dogs, moderate lameness in one dog, moderate lameness with intermittent skipping in seven dogs, intermittent skipping and shortened stride in six dogs, and postural change in one dog (narrow stance).

#### Palpation Findings

Most of the dogs had multiple abnormal palpation findings within the lumbar and/or hind limbs of the affected sciatic nerve: 22/29 dogs (76%) had pain, spasm, or trigger points in the iliopsoas muscle; 10/29 (35%) showed restriction or pain on hip extension. One of these dogs also experienced pain during hip flexion; 9/29 (31%) had pain or tenderness on palpation of the lumbosacral area; 14/29 (48%) presented with pain on direct palpation of the gluteal/sciatic area or restriction on the piriformis stretch. Nineteen 19/29 dogs (66%) also presented with abnormal findings on the stifle. Findings included effusion and/or pain on extension or flexion of the stifle or luxating patella; 4/29 (14%) had rotation or malalignment at the sacroiliac (SI) joint, and one 1/29 dog (3%) had effusion on the tarsal joint. Most of the dogs had abnormal findings in multiple anatomical areas of the affected limb. One dog exhibited abnormal palpation in the gluteal area without any other abnormal clinical findings (See Table 1).

**Table 1:** The top portion contains descriptions of the abnormal palpation findings (pain, discomfort, warmth, and effusion) during the orthopedic evaluation.

**Abnormal examination findings case nr:**	**Hip extension**	**Hip flexion**	**Lumbosacral palpation**	**Iliopsoas palpation**	**Stifle**	**Palpation of the gluteal area or piriformis stretch**	**SI joint rotation/** **malalignment**	**Tarsus**	**Left/Right leg**
1	X			X		X	X		R
2				X			X		R
3			X	X		X	X		L
4	X		X	X	L				R
5			X	X	X				L
6				X	X	X			R
7			X						L
8				X		X			R
9	X	X							L+R
10	X				X	X			R
11			X	X	X	X			R
12						X			L
13	X		X	X	X	X			R
14					X				L
15			X	X	X				L
16	X			X	X	X			R
17	X				X	X			L
18						X			L
19				X	X				L
20				X					L
21	X			X	X				L
22				X	X				R
23	X		X	X	X	X	X		L
24			X	X	X	X			R
25	X			X	X	X			R
26					X				R
27				X	X				R
28				X	X				L
29				X				X	R

*L, Left hind leg; R, Right hind leg*.

### Ultrasound Findings

#### The Appearance of the Sciatic Nerves

**Figure 3:**
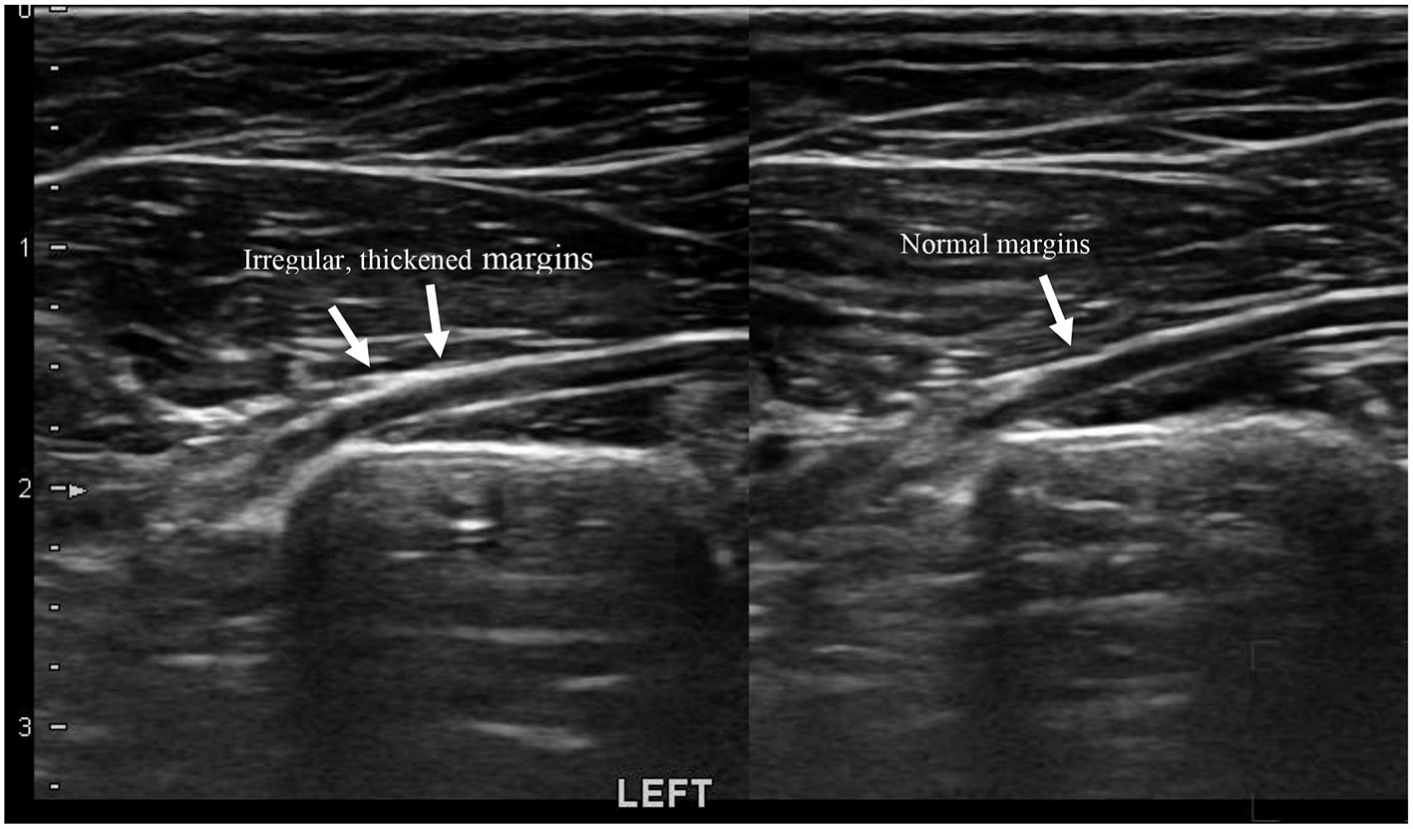
Linear image of a sciatic nerve with neuritis. There are irregular thickened margins noted on the left sciatic nerve.

The sciatic nerves were examined on longitudinal axis. The non-affected sciatic nerve of each dog was used as a normal reference for that individual. 28/29 (96%) dogs had non-affected sciatic nerve on the ipsilateral side, one dog (Case no: 9) had bilateral findings. The sciatic nerve was considered normal if it appeared as a hypoechoic tubular structure with paraller echogenic lines within and possessed sharply marginated hyperechoic borders. Findings were in concordance with what has been described in the literature (14, 27). The affected sciatic nerves were also hypoechoic tubes with hyperechoic borders and appeared enlarged, with irregular and thickened borders. This seemed to be a relatively consistent finding throughout the study, as 22 out of the 29 dogs (76%) had irregularity of the margins (See Figure 3).

#### Measurements of the Sciatic Nerves

In 29 dogs, measurement of the inner diameter of unaffected and affected sciatic nerves was performed at the site of the gemelli muscles. Measurements were taken from longitudinal ultrasound images of the nerves. The inner diameter of the unaffected nerve ranged from 0.07 to 1.9 mm with a mean ± SD of 1.2 ± 0.32 mm. The inner diameter of the affected nerve ranged from 0.09 to 2.1 mm, with a mean ± SD of 1.37 ± 0.37 mm. The inner diameter of the sciatic nerve on the affected side was significantly larger than the contralateral (unaffected) side (*p* < 0.00001). In 27 of these dogs, we also measured the outer sciatic diameter of both unaffected and affected sciatic nerves, over the gemelli muscles. The outer diameter of the unaffected nerve ranged from 1.6 to 2.8 mm, with a mean ± SD of 2.14 ± 0.265 mm. The outer diameter of the affected nerve ranged from 1.9 to 3.1 mm, with a mean ±SD of 2.4 ± 0.310 mm. The outer diameter of the sciatic nerve on the affected side was significantly larger than the contralateral (unaffected) side (*p* < 0.00001).

For each individual dog the percentage change in sciatic nerve diameter between the affected and unaffected sides was calculated. The percent difference in sciatic nerve inner diameter ranged from −8 to 29%, with a mean ± SD of 15 ± 14.66%. The sciatic nerve on the affected side was more prominent in 26/29 cases. The percent difference in sciatic nerve outer diameter ranged from 0 to 29%, with a mean ± SD of 12 ± 7.71% (See Table 2).

**Table 2:** Ultrasonographic findings and measurements of the sciatic nerves.

**Case no**.	**Breed**	**Normal inner diameter (mm)**	**Affected inner diameter (mm)**	**Inner diameter difference (percentage)**	**Normal outer diameter (mm)**	**Affected outer diameter (mm)**	**Outer diameter difference (percentage)**	**Irregularity of the margins in affected side Yes/No**
1	Border Collie	1.3	1.2	−0.1 (−8%)	2.2	2.2	0 (0%)	Yes
2	Australian Shepherd	1.3	1.2	−0.1 (−8%)	2.2	2.3	0.1 (5%)	Yes
3	Standard Poodle	1.3	1.4	0.1 (15%)	2.3	2.4	0.1 (4%)	No
4	Boxer	1.1	1.2	0.1 (9%)	2.0	2.0	0 (0%)	No
5	Golden Retriever	1.2	1.3	0.1 (8%)	2.3	2.7	0.4 (17%)	No
6	Border Collie	1.2	1.4	0.2 (15%)	2.0	2.3	0.3 (15%)	Yes
7	Border Collie	1.2	1.5	0.3 (25%)	2.1	2.8	0.7 (19%)	Yes
8	Shetland Sheepdog	1.2	1.4	0.2 (17%)	2.0	2.3	0.3 (15%)	Yes
10	Border Collie	1.3	1.4	0.1 (8%)	2.3	2.4	0.1 (4%)	Yes
11	Border Collie	1.6	1.7	0.1 (6%)	1.7	2.0	0.3 (18%)	Yes
12	Border Collie	1.3	1.6	0.3 (23%)				Yes
13	Border Collie	1.1	1.2	0.1 (9%)	2.0	2.1	0.1 (5%)	Yes
14	Border Collie	1.8	2.0	0.2 (11%)	2.8	3.1	0.3 (10%)	Yes
15	Rat Terrier	0.07	0.09	0.02 (29%)	1.6	1.9	0.3 (19%)	Yes
16	Collie	1.1	1.2	0.1 (9%)	2.3	2.6	0.3 (13%)	Yes
17	German Shepherd	1	1	0 (0%)	2.3	2.8	0.5 (22%)	Yes
18	Jack Russel Terrier	1	1.4	0.4 (40%)	2	2.3	0.3 (15%)	Yes
19	Border Collie	1.2	1.3	0.1 (8%)	2.1	2.7	0.6 (29%)	Yes
20	Border Collie	1.2	1.6	0.4 (33%)	2.0	2.3	0.3 (15%)	Yes
21	Mix Breed	1.1	1.2	0.1 (9%)	2.1	2.7	0.6 (28%)	Yes
22	Nova Scotia Duck Tolling Retriever	1.3	2.1	0.8 (61%)	2.1	2.4	0.3 (14%)	Yes
23	Border Collie	0.9	1.2	0.3 (33%)	2.0	2.1	0.1 (5%)	Yes
24	Labrador Retriever	1.9	1.9	0 (0%)	2.7	2.8	0.1 (4%)	Yes
25	Staffordshire Bull Terrier	1.0	1.1	0.1 (10%)	2.2	2.3	0.1 (5%)	No
26	German Shepherd	1.5	1.7	0.2 (13%)	2.4	2.6	0.2 (8%)	Yes
27	Leonberger	1.3	1.6	0.3 (23%)	2.3	2.6	0.3 (13%)	No
28	Border Collie	1.0	1.2	0.2 (20%)	1.7	1.9	0.2 (12%)	Yes
29	Whippet	1.1	1.2	01 (9%)	2.0	2.2	0.2 (10%	No

*Case no. 9 had abnormal bilateral changes in the sciatic nerves, and thus was not included in the chart*.

When comparing dogs with underlying orthopedic diagnosis against dogs with no underlying orthopedic diagnosis, there was no significant difference in sciatic nerve diameter in either limb, nor in the percent difference in sciatic nerve diameter.

#### Ultrasound Findings of the Piriformis Muscle and Tendon

**Figure 4:**
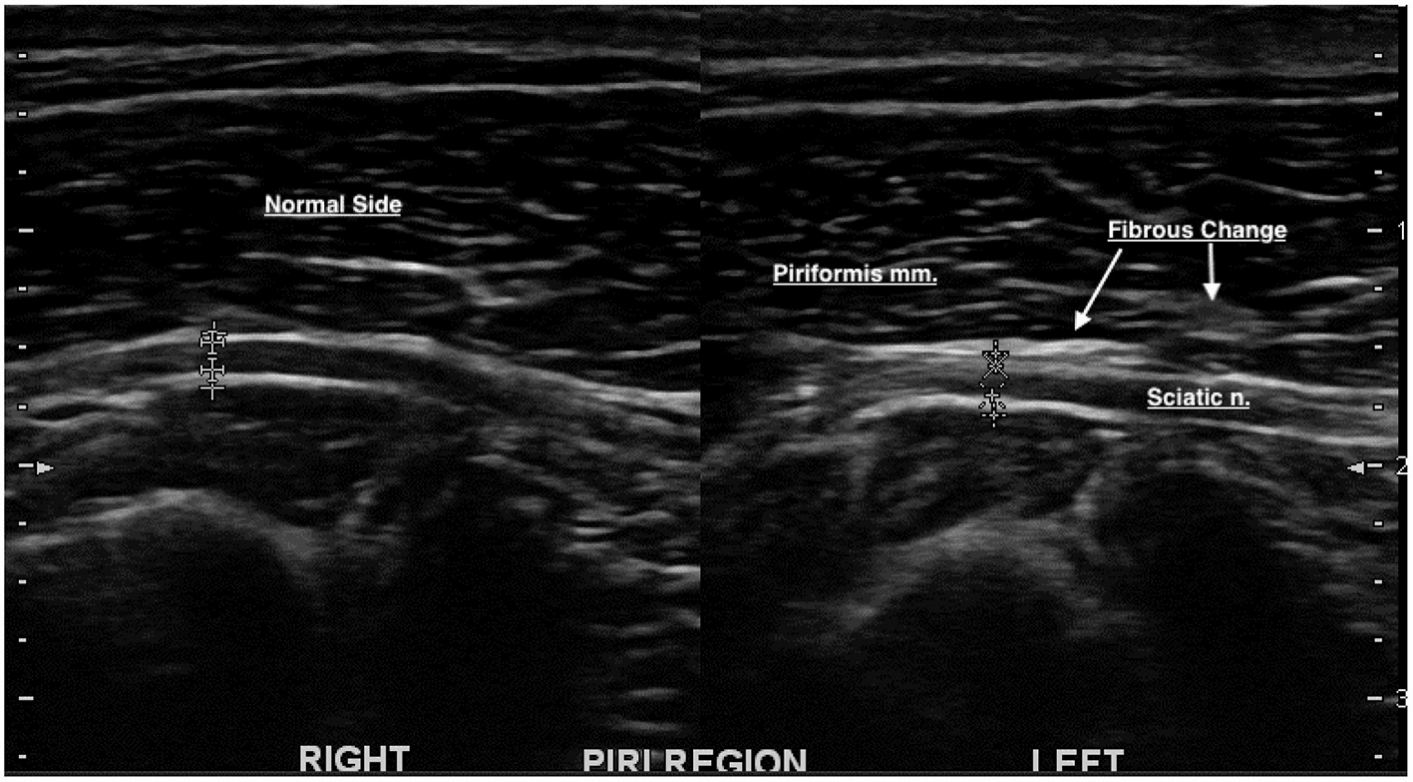
Increased echogenicity/hyperechoic change was noted on the deep piriformis margin on the left side, superficial to the sciatic nerve. Fibrous impingement of the sciatic nerve was also noted.

The piriformis muscle was evaluated from its deep margin, superficial to the sciatic nerve, onto its tendinous attachment to the greater trochanter. Echogenicity, homogeneity, size, and possible impingement with the sciatic nerve were evaluated (See Figure 4). Increased echogenicity was observed in 13/29 (45%) dogs, decreased echogenicity in 4/29 (14%) dogs, normal echogenicity in 11/29 (38%) dogs, muscle atrophy in 5/29 (17%) dogs, and enthesiophytes in the insertion of the tendon to the greater trochanter in 3/29 (10%) dogs. Impingement of the sciatic nerve was also noted in 6/29 (21%) dogs (See Table 3). The muscle was considered atrophied if there was a hyperechoic and condensed muscle fiber pattern.

**Table 3:** Ultrasonographic findings of the piriformis muscles and tendons on the affected side.

**Case no**.	**Ultrasonographic findings piriformis muscle, affected side**	**Impingement to sciatic nerve Yes/No**
1	Increased echogenicity	No
2	Increased echogenicity, fibrous fiber pattern	Yes
3	Normal echogenicity in the muscle belly, Increased echogenicity at the insertion of the tendon	No
4	Normal echogenicity, significant muscle atrophy	No
5	Increased echogenicity	No
6	Normal fiber pattern and echogenicity	No
7	Increased echogenicity	No
8	Increased echogenicity, fibrous fiber pattern	Yes
9	Normal fiber pattern and echogenicity	No
10	Normal fiber pattern and echogenicity at the muscle, small enthesiophytes at the tendon insertion	No
11	Increased echogenicity	No
12	Increased echogenicity at piriformis muscle, loss of detail at the tendon insertion	No
13	Decreased echogenicity at both muscle and tendon	No
14	Normal fiber pattern and echogenicity	No
15	Decreased echogenicity, mild loss of fiber detail, thickening in the muscle belly	Yes, mild
16	Normal fiber pattern and echogenicity at the muscle, small enthesiophytes at the tendon insertion	Yes, mild
17	Increased echogenicity	Yes
18	Normal fiber pattern and echogenicity	No
19	Increased echogenicity, fibrous fiber pattern	No
20	Normal fiber pattern and echogenicity at the muscle, small enthesiophytes at the tendon insertion	No
21	Increased echogenicity, fibrous fiber pattern	No
22	Normal fiber pattern and echogenicity	No
23	Increased echogenicity with hypoechoic foci, increased thickness at the tendon	Yes
24	Normal fiber pattern and echogenicity at the muscle, small enthesiophytes at the tendon insertion	No
25	Normal fiber pattern and echogenicity	No
26	Mildly increased echogenicity, normal fiber pattern	No
27	Mildly increased echogenicity	No
28	Normal fiber pattern and echogenicity	No
29	Normal fiber pattern and echogenicity. Mild atrophy	No

#### Ultrasound Findings of Other Deep Gluteal Muscles

Ultrasonographic findings of other deep gluteal muscles/tendons surrounding the proximal sciatic nerve were also detected. These included the gemelli muscle, internal obturator tendon and bursa, and middle gluteal muscle, and insertional tendon. The echogenicity of the muscles and tendons, homogeneity of the muscles and tendons, changes in the internal obturator tendon bursa, and osteophytes at the middle gluteal tendon insertion site/enthesis were evaluated and recorded.

**Figure 5:**
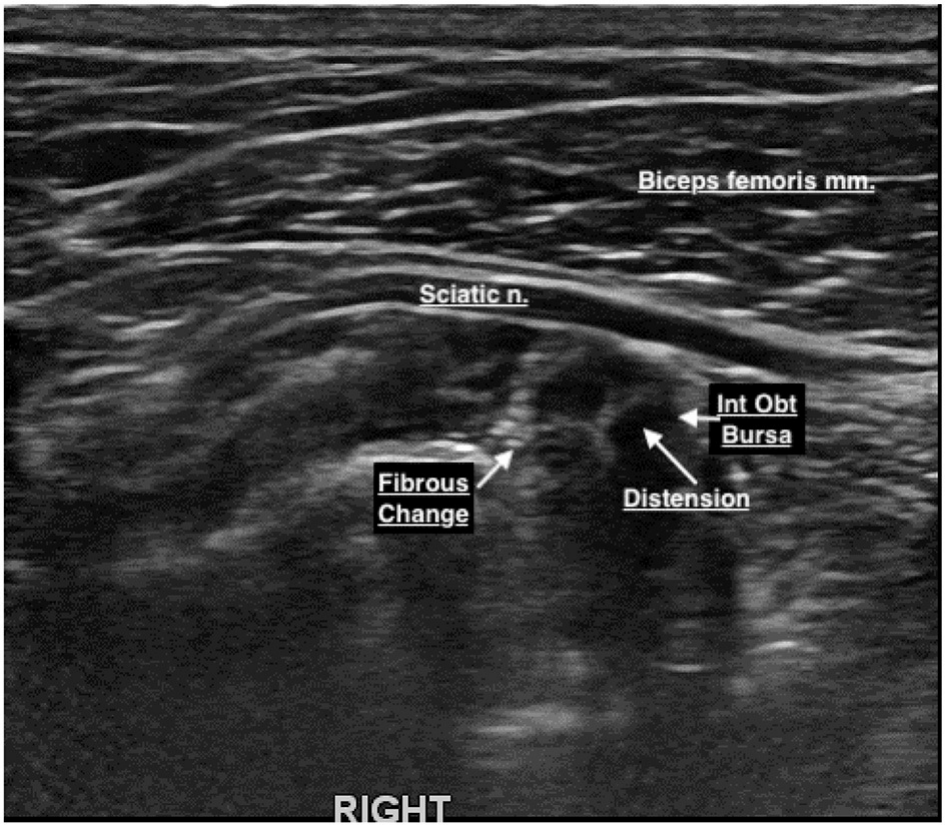
Internal obturator bursa inflammation and fibrous change.

There was increased echogenicity in 15/29 (52%) gemelli muscles, 8/29 (28%) obturator muscles, and 2/29 (7%) middle gluteal muscles on the affected side. Hypoechoic changes were noted in 2/29 (7%) of the gemelli muscles and 3% of the obturator muscle. Muscle atrophy was noted in 4/29 (14%) gemelli muscles and 1/29 (3%) middle gluteal muscle of the affected side. Enthesiophytes were noted in 6/29 (21%) of the middle gluteal muscle tendons at their attachment to the greater trochanter (See Table 4 and Figure 5).

**Table 4:** Ultrasonographic findings of the internal obturator, gemelli and middle gluteal muscles, affected side.

**Case no:**	**Internal obturator muscle, tendon, and its bursa**	**Gemelli muscles**	**Middle gluteal muscle and its tendon**
1	Increased echogenicity at internal obturator tendon/bursal region		–
2	–	Increased echogenicity	–
3	Increased echogenicity	Increased echogenicity	Normal fiber pattern and echogenicity
4	Increased echogenicity	Increased echogenicity	Normal fiber pattern and echogenicity
5	Bursal inflammation at the tendon insertion site	Focal areas with increased echogenicity	Normal fiber pattern and echogenicity
6	–	Normal fiber pattern and echogenicity	–
7	–	Increased echogenicity	–
8	–	Increased echogenicity, loss of detail in fiber pattern	Increased echogenicity
9	Increased echogenicity	Increased echogenicity	Normal fiber pattern and echogenicity
10	Decreased echogenicity, bursal inflammation	Increased echogenicity	Normal fiber pattern and echogenicity
11	–	Increased echogenicity	Normal fiber pattern and echogenicity
12	–	Increased echogenicity	–
13	Increased echogenicity	Increased echogenicity	–
14	–	Increased echogenicity	–
15	–	Decreased echogenicity	Normal fiber pattern and echogenicity in the muscle, prominent enthesiophytes at the insertion onto greater trochanter
16	Normal fiber pattern and echogenicity	Decreased echogenicity	Normal fiber pattern and echogenicity in the muscle, mild enthesiophytes at the insertion onto greater trochanter
17	–	Increased echogenicity, loss of detail in fiber pattern	Normal fiber pattern and echogenicity
18	Normal fiber pattern and echogenicity	Normal fiber pattern and echogenicity	Normal fiber pattern and echogenicity
19	–	Increased fiber pattern	Increased echogenicity
20	Normal fiber pattern and echogenicity	Decreased echogenicity	–
21	–	Normal fiber pattern and echogenicity	–
22	–	Decreased echogenicity	Normal fiber pattern and echogenicity in the muscle, small enthesiophyte at the insertion onto greater trochanter
23	Increased echogenicity	Increased echogenicity	–
24	–	Decreased echogenicity	Normal fiber pattern and echogenicity
25	Increased echogenicity	Increased echogenicity	Normal fiber pattern and echogenicity
26	Normal fiber pattern and echogenicity	Normal fiber pattern and echogenicity	Normal fiber pattern and echogenicity in the muscle, small enthesiophyte at the insertion onto greater trochanter
27	–	Normal fiber pattern and echogenicity	Normal fiber pattern and echogenicity
28	–	Decreased echogenicity	Normal fiber pattern and echogenicity in the muscle, prominent enthesiophytes at the insertion onto greater trochanter
29	Increased echogenicity	Normal fiber pattern and echogenicity	Normal fiber pattern and echogenicity in the muscle, small enthesiophyte at the insertion onto greater trochanter

*–: Findings were not commented on the ultrasound report*.

### Other Diagnostic Imaging Modalities and Their Findings

Radiographic imaging was performed in 24/29 dogs with examination findings suggestive of either stifle or lumbosacral disease. Radiological findings were as follows: 11/29 (38%) had mild stifle effusion, 5/29 (17%) had moderate stifle effusion, 2/29 (7%) had stifle arthritis, 3/29 (10%) had remodeling in the lumbosacral space, 2/29 (7%) had hip arthritis, 1/29 (3%) had lumbosacral spondylosis, and 1/29 (3%) had transitional lumbar vertebrae.

Magnetic resonance imaging of the lumbar spine was performed in 10 dogs with examination findings and radiographs suggestive of underlying lumbosacral disease. The MRI findings included narrowing of the foramina of the L6-7 nerve roots (*n* = 2), lumbosacral disc disease with suspicion of dynamic compression (*n* = 2), mild LS disc herniation without compression (*n* = 3), and normal findings (*n* = 3).

### Diagnosis

9/29 (29%) dogs had primary sciatic neuritis without any underlying disease detected on orthopedic examination or diagnostic imaging. There were 7/29 (24%) dogs with lumbosacral disease, 7/29 (24%) dogs with stifle disease on the ipsilateral side, 2/29 (7%) dogs with hip arthritis on the ipsilateral side, 1/29 (3%) dog with both hip and stifle arthritis on the ipsilateral side, 2/29 (7%) dogs with iliopsoas disease on the ipsilateral side, and 1/29 (3%) dog with tarsal disease on the ipsilateral side.

## Discussion

Ultrasound was performed of the proximal part of the sciatic nerve and the surrounding muscles in the deep gluteal region. In the longitudinal image, the normal sciatic nerve of a dog appears as a hypoechoic tubular structure with paraller echogenic linear structures within and sharply marginated hyperechoic borders on both sides of the hypoechoic tubular structure (14, 27). The sciatic nerve findings of the affected nerves in this study were consistent. The abnormal ultrasonographic findings in the sciatic nerves showed either an increased thickness or irregular borders of the nerve, or both, compared to the contralateral side. This was consistent with the findings from human nerve ultrasound studies, indicating neuritis (18–21, 23). The average percent difference in sciatic nerve diameter between the unaffected and affected sides was 15% for the inner diameter and 12% for the outer diameter. The sciatic nerve of the affected limb was significantly larger than that of the unaffected limb, consistent with neuritis.

We also investigated whether dogs with underlying orthopedic disease would have larger increases in sciatic nerve diameter due to clinical disease severity, in comparison to dogs with ultrasonographic evidence of sciatic neuritis but no clinical evidence of underlying orthopedic disease. There was no significant difference in sciatic nerve measurements between these two groups, suggesting that the underlying reason for the sciatic neuritis does not significantly affect the measurements nor the extent of the swelling of the nerve. Allthough there was no underlying orthopedic disease diagnosed, there was ultrasonographic changes recorded within the deep gluteal muscles with 7/9 (78%) of these dogs. Allthough the numbers of the dogs without an underlying orthopedic disease is low (9/29; 30%) this is an interesting fact, and it would be interesting to have future studies concentrating on relationship with deep gluteal changes and sciatic neuritis only.

A human imaging study used nerve-root anesthesia, which suggested that nerve swelling had an 80% positive predictive value for radicular pain (29). In our study, 14 (48%) of the 29 dogs had pain in the sciatic region on physical examination. There could also be a clinical correlation between nerve swelling and radicular pain in dogs. However, the evaluation of radicular pain in dogs is highly subjective and challenging. Therefore, further investigation is necessary on this subject.

We also performed an ultrasound of the muscles in the deep gluteal region around the sciatic nerve to evaluate the possible correlation between changes within the sciatic nerve and the surrounding muscles. Muscles examined included the piriformis muscle, gemelli muscles, middle gluteal muscle, and internal obturator muscle. The normal appearance of the muscles in the longitudinal image is hypoechoic, with multiple hyperechoic longitudinal or oblique strides within the muscle belly due to connective tissue surrounding the muscle fascicles. The epimysium surrounding the muscle bundle group is seen as slightly thicker hyperecoic line. In transverse imaging these hyperechoic connective tissues are seen as small dots within the hypoechoic muscle belly (30–32). In atrophied muscles, the hyperechoic fascia becomes more prominent, and the hypoechoic water and protein-rich muscle bundles become smaller, resulting in increased echogenicity (32).

In a study on ultrasonographic findings in humans with piriformis syndrome, it was reported that the piriformis muscles on the symptomatic side were significantly larger than those on the asymptomatic side. Increased echogenicity within the piriformis muscles of symptomatic patients was also reported (3). In our study, atrophy was noted in 5 of the 29 piriformis muscles (17%). Muscles were considered atrophied if there was a hyperechoic and condensed fiber pattern (cases no. 1, 4, 10, 23, and 24). In these canine patients, there was generalized atrophy within the thigh and deep gluteal muscles on that side. Generalized muscle atrophy was most likely due to underlying orthopedic issues. The diagnoses within these canine patients were as follows: lateral foraminal stenosis, osteoarthrosis of the hip joint, post-TPLO sciatic neuritis, lateral foraminal stenosis, and partial cranial cruciate tear. Our findings in dogs were not concordant with the ultrasonography findings in humans with piriformis syndrome, which showed increased muscle mass of the piriformis muscle. Impingement between the piriformis muscle and sciatic nerve was also noted in 6/29 (21%) dogs. The impingement was due to increased fibrous tissue within the piriformis muscle in the area of contact between the sciatic nerve and the muscle.

Abnormal ultrasonographic findings were observed in the gemelli muscles in 20/29 (67%) of cases. Fifteen 15/29 (52%) canine patients had increased echogenicity and 5/29 (17%) had decreased echogenicity. Increased echogenicity is an indication of muscle atrophy. The function of the gemelli muscle is to laterally rotate the hip joint, along with preventing medial rotation of the hip joint on weight-bearing. Hyperechoic muscle fiber findings indicated muscle atrophy. Among the 15 dogs with hyperechoic findings of the gemelli muscles, there were five dogs with primary sciatic neuritis without underlying diseases, five with lumbosacral disease, three with hip osteoarthritis, one with stifle disease, and one with iliopsoas enthesitis. In dogs suffering from hip disease, lumbosacral disease, or primary sciatic neuritis, atrophy may be due to a restriction of the movement of the gemelli muscles. Anatomically, the sciatic nerve runs superficial to and innervates the gemelli muscle (see Figure 1). Considering all the abovementioned factors, the authors believe that the ultrasonographic findings of the gemelli muscle in this study are most likely due to direct irritation and functional impairment secondary to sciatic neuritis or underlying diseases.

There were abnormal ultrasonographic findings in one 1/29 (3%) middle gluteal muscle, which was atrophied (case 24, partial cranial cruciate ligament tear) and four 4/29 (14%) middle gluteal tendon insertions (cases 8, 26, 28, 29). The middle gluteal muscle performs extension of the hip joint and medial rotation of the hip. This indicates that despite the location in the deep gluteal area, the middle gluteal muscle may not play a role in sciatic neuritis in dogs, and concurrent pathology is not expected.

Abnormal ultrasonographic findings were observed in 9/29 (31%) internal obturator muscles. Increased echogenicity and fibrous tissue were noted in all nine muscles, indicating muscle atrophy. Changes were more prominent at the internal obturator tendon insertion site. The function of the internal obturator muscle is to laterally rotate the hip, and the sciatic nerve innervates the muscle. Of these nine dogs with abnormal ultrasonographic findings in the internal obturator muscles, only five were diagnosed with sciatic neuritis. Thus, despite the close anatomical relationship between the internal obturator muscle and sciatic nerve, there was no correlation between sciatic neuritis and increased echogenicity and fibrous tissue of the internal obturator muscle.

Stretching or impingement of a nerve is the most common underlying cause of neuritis. This also seems to be the case in this study. In our study, lumbosacral disease was one of the most frequent concurrent underlying conditions in dogs diagnosed with sciatic neuritis. In stifle disease, dogs often offload the affected leg by hip flexion, abduction, and external rotation. This may cause prolonged stretching of the sciatic nerve and, in time, neuritis.

The fact that agility dogs were overrepresented (7/9; 78% dogs with LS disease were agility dogs) with sciatic problems may be because agility is a very physically demanding sport, thus predisposing dogs to various injuries. In sports that include jumping, especially agility, dogs extend their hip joints at high speed, predisposing the lumbosacral area to high biomechanical forces. Piriformis and middle gluteal muscles act as extensors of the hip joint. The piriformis muscle contracts when dogs extend their hip while jumping. The sciatic nerve running deep to the piriformis muscle may explain the commonly found irritation of the nerve. The authors suspect that this dynamic biomechanical relationship may also be an additional predisposing factor for sciatic neuritis in sporting dogs.

Seven 7/29 (24%) dogs had hind limb skipping as the primary clinical sign. There is an ongoing discussion in the veterinary field about the causes of skipping in the hind limbs of dogs, some of which include medial patellar luxation and superficial digital flexor tendon luxations. There is also discussion on the possible neural causes of hind limb skipping. The possible correlation between skipping and sciatic neuritis is beyond the scope of this study. Nevertheless, we believe it might be helpful to perform an ultrasound of the sciatic nerve and deep gluteal area for the canine patients with hindlimb skipping, as they may also have sciatic neuritis with or without other underlying issues.

To the best of our knowledge, this is the first study documenting ultrasonography findings in a case series of the sciatic nerve and deep gluteal muscles and their pathology in dogs. Ultrasound appears to be a useful tool for assessing neuritis in humans and should be considered for the assessment of neuritis in dogs. Ultrasonography may also be beneficial for visualizing the response to treatment in many cases, based on a decrease in nerve enlargement (18). There should also be more in-depth investigations on primary sciatic neuritis and its clinical signs in dogs.

Blood flow was found to be decreased in the sciatic nerve when the hip was both flexed and internally rotated in a study with 20 dogs (33). This standing position is typical for dogs with hip dysplasia. Therefore, dogs with hip dysplasia are predisposed to sciatic neuritis, and this should be considered when examining, treating, and rehabilitating dogs with hip dysplasia. In our study, two 2/29 (7%) dogs were diagnosed with arthritis of the hip on the ipsilateral side of the sciatic neuritis.

There were also some limitations to this study. The diagnosis of the sciatic neuritis was established based on the ultrasonography findings. It would have been interesting to compare ultrasonographic changes to the results of nerve biopsies. However this was a retrospective case study, and at the time of the diagnosis the clinical signs and the ultrasonographic findings supported the diagnosis of the neuritis. The ultrasonography findings were also in concordance with the human studies of ultrasonography findings of neuritis (18–21, 23). In addition, the sample size in this study is limited (n: 29). However, the differences between the measurements of the affected and unaffected nerves were found to be significant.

Ultrasonography of the nerves is performed in two planes in humans, scanning the nerve in both the long and short axes. In humans, the short axis is preferred especially in the carpal tunnel area, due to serpentine-like routing of the nerves. In our study, we examined and measured the sciatic nerves only along the long axis, based on the familiarity and experience of the ultrasonographer in our study. The proximal part of sciatic nerve in a dog travels mainly in a straight line and is easy to follow along the long axis. The measurements were performed from the linear axis as in a previous study in dogs (27).

Neurology specialists commonly use electrodiagnostics in canine patients suspected of having neuritis. Electrodiagnostics do not provide spatial information about the surroundings of the nerve, which could help determine the cause of the neuritis, further assisting with decision-making regarding the treatment. This study concentrated on the ultrasound findings of the deep gluteal muscles and sciatic nerves. No electrodiagnostics were performed on the canine patients in this study. In the future, it would be intriguing to see the possible correlation between electrodiagnostic testing and ultrasonography findings in dogs with suspected sciatic neuritis.

The piriformis stretch was performed as described in a previous study (34). The muscle is stretched in flexion, external rotation, and adduction of the hip joint. Hip dysplasia is relatively common in dogs, and manipulating the hip joint may be uncomfortable for some dogs. In addition, the stretch may be faulted in dogs because they are quadruped and are incapable of outwardly rotating the stifle, hence the lack of consistency of the stretch being performed correctly. Therefore, the findings regarding the piriformis stretch in the study were interpreted together with other clinical findings.

Another factor to be considered is the possibility of anatomical variants in dogs. Recognition of anatomical variants and detailed knowledge of the areas in which entrapment is most likely to occur enhances the accuracy of imaging evaluation. To the best of our knowledge, there are no studies in dogs investigating the anatomical variants within the sciatic nerve. Ultrasound examination was performed under the assumption that there were no such variants in the dog. No anatomical variants were found in the canine patients of this study.

The purpose of this study was to report and discuss ultrasonography findings in the proximal sciatic nerve and deep gluteal muscles in dogs with suspected sciatic neuritis. According to our study findings, ultrasound imaging can be considered a valuable tool for detecting changes in the sciatic nerve. There were also changes in the deep gluteal muscles. Although the connection between sciatic neuritis and the changes within the deep gluteal muscles is still unclear, we recommend including ultrasonography of the piriformis muscle, gemelli muscles, internal obturator muscle, and tendons in the ultrasound examination of the sciatic nerve in dogs to obtain more information on the pathology and prognosis of disease, rendering rehabilitation planning more precise and appropriate.

## Data Availability Statement

The data analyzed in this study is subject to the following licenses/restrictions: patient data including ultrasound images and reports may be provided should it be requested. Requests to access these datasets should be directed to ttoijala@gmail.com.

## Ethics Statement

The animal study was reviewed and approved by VOSM Research Committee. Written informed consent was obtained from the owners for the participation of their animals in this study.

## Author Contributions

TT collected the patient data, participated in most of the orthopedic and ultrasound examinations, and wrote the manuscript. DC performed the ultrasound examinations and contributed to the writing and editing of the manuscript. SC performed the orthopedic examinations and contributed to the writing and editing of the manuscript. All authors contributed to the article and approved the submitted version.

## Conflict of Interest

The authors declare that the research was conducted in the absence of any commercial or financial relationships that could be construed as a potential conflict of interest.

## Publisher's Note

All claims expressed in this article are solely those of the authors and do not necessarily represent those of their affiliated organizations, or those of the publisher, the editors and the reviewers. Any product that may be evaluated in this article, or claim that may be made by its manufacturer, is not guaranteed or endorsed by the publisher.
